# Development and Inter-Laboratory Validation of Unlabeled Probe Melting Curve Analysis for Detection of JAK2 V617F Mutation in Polycythemia Vera

**DOI:** 10.1371/journal.pone.0026534

**Published:** 2011-10-20

**Authors:** Zhiyuan Wu, Hong Yuan, Xinju Zhang, Weiwei Liu, Jinhua Xu, Wei Zhang, Ming Guan

**Affiliations:** 1 Department of Laboratory Medicine, Huashan Hospital, Shanghai Medical College, Fudan University, Shanghai, People's Republic of China; 2 Department of Clinical Laboratory, The First Affiliated Hospital of Dalian Medical University, Dalian, People's Republic of China; 3 Central Laboratory, Huashan Hospital, Shanghai Medical College, Fudan University, Shanghai, People's Republic of China; 4 Department of Dermatology, Huashan Hospital, Shanghai Medical College, Fudan University, Shanghai, People's Republic of China; 5 Shenzhen Key Laboratory for Translational Medicine of Dermatology, Shenzhen-PKU-HKUST Medical Center, Shenzhen, People's Republic of China; University of Houston, United States of America

## Abstract

**Background:**

JAK2 V617F, a somatic point mutation that leads to constitutive JAK2 phosphorylation and kinase activation, has been incorporated into the WHO classification and diagnostic criteria of myeloid neoplasms. Although various approaches such as restriction fragment length polymorphism, amplification refractory mutation system and real-time PCR have been developed for its detection, a generic rapid closed-tube method, which can be utilized on routine genetic testing instruments with stability and cost-efficiency, has not been described.

**Methodology/Principal Findings:**

Asymmetric PCR for detection of JAK2 V617F with a 3′-blocked unlabeled probe, saturate dye and subsequent melting curve analysis was performed on a Rotor-Gene® Q real-time cycler to establish the methodology. We compared this method to the existing amplification refractory mutation systems and direct sequencing. Hereafter, the broad applicability of this unlabeled probe melting method was also validated on three diverse real-time systems (Roche LightCycler® 480, Applied Biosystems ABI® 7500 and Eppendorf Mastercycler® ep realplex) in two different laboratories. The unlabeled probe melting analysis could genotype JAK2 V617F mutation explicitly with a 3% mutation load detecting sensitivity. At level of 5% mutation load, the intra- and inter-assay CVs of probe-DNA heteroduplex (mutation/wild type) covered 3.14%/3.55% and 1.72%/1.29% respectively. The method could equally discriminate mutant from wild type samples on the other three real-time instruments.

**Conclusions:**

With a high detecting sensitivity, unlabeled probe melting curve analysis is more applicable to disclose JAK2 V617F mutation than conventional methodologies. Verified with the favorable inter- and intra-assay reproducibility, unlabeled probe melting analysis provided a generic mutation detecting alternative for real-time instruments.

## Introduction

Somatic mutations of the JAK2 gene at V617F, which leads to constitutive JAK2 phosphorylation and kinase activation, occurs in almost all cases of polycythemia vera and approximately 50% of cases of chronic idiopathic myelofibrosis [1]–[4]. Furthermore, diagnostic criteria for polythemia vera (PV), essential thrombocytosis, and primary myelofibrosis were revised by incorporating this recently described molecular marker [5], [6]. Because identification of the V617F mutation will be of potential use in the diagnosis, prognosis, and perhaps selection of treatment for myeloproliferative neoplasmas, many tools have been developed to genotype JAK2 V617F, including techniques such as restriction fragment length polymorphism (RFLP) [2], amplification refractory mutation system (ARMS) [7], real-time PCR [8], [9] and direct sequencing [10], [11]. However, some of these methods have limitations in sensitivity or in cost. Furthermore, screening assays for the JAK2 mutation are not standardized and the possibility of false negative or false positives can be real especially when low mutant allele burden in the peripheral blood need to be detected [12], [13]. There is now an urgent need for a quick, cost-efficient and reliable assay to identify the mutation.

Recently, unlabeled probe melting curve assay (MCA) with saturating DNA dye for genotyping has been described [14]–[16]. Unlabeled probe MCA is a closed-tube, homogeneous method for genotyping without fluorescently labeled probes or allele specific PCR. The method combines both saturate dyes and unlabeled oligonucleotide probes in an asymmetric PCR, leading to simultaneous production of probe-target and whole amplicon double-stranded DNA duplexes that can be analyzed from the same PCR run and would be suitable for genotyping. Melting curve methods have now been adapted to real-time PCR instruments, and compared to sequencing or allele-specific PCR, represent high throughput and cost saving methods with the further advantage of reducing post-PCR handling of PCR products [17], [18].

Unlabeled probe MCA technology has been used for human single nucleotide polymorphism (SNP) genotyping [19], [20], mutation detection [21] and bacterial species identification [22]. However, instruments vary widely in their ability to genotype variants for melting analysis [23], [24] in clinical laboratories. The ability to differentiate complex melting species depends on the quality of the melting curve generated. Understanding the melting capabilities of these instruments will guide the appropriate use of this technology.

Since unlabeled probe technology could be a rapid and convenient tool for detecting the various JAK2 V617F mutations, we decided to establish one such method with the prerequisite that it should be reliable enough to give similar results on common instruments. We first develop a simple and sensitive method for detecting the V617F mutation in the JAK2 gene via unlabeled probe MCA in Rotor-Gene® Q (Qiagen, Valencia, CA) and validate the method on three other real-time instruments at two different sites.

## Materials and Methods

### Patients

Forty blood samples were obtained from polycythemia vera patients seen in the Huashan Hospital of Fudan University and the First Affiliated Hospital of Dalian Medical University. Written informed consents were received from all participants. DNA was extracted from blood samples collected in ethylenediamine tetraacetic acid anticoagulant with the QiaAmp® DNA Blood Mini kit according to manufacturer's directions (Qiagen, Valencia, CA). Homozygous mutant (JAK2 V617F/JAK2 V617F) human erythroleukemia (HEL) and homozygous wild type multiple myeloma (RPMI8226) cell lines were purchased from the cell bank of type culture collection of Chinese Academy of Sciences and used as positive and negative controls, respectively. In compliance with Helsinki Declaration of 1975 as revised in 1996, this study was approved by the Institutional Review Board of Huashan Hospital.

### Genotyping with unlabeled probe melting curve assay

Genotyping of JAK2 V617F was developed by unlabeled probe high resolution melting assay in the Rotor-Gene® Q real-time PCR system. Primer and probe sequences used for PCR are listed in Table 1. To prevent the extension of the probe during PCR, a 3′- carbon based C3 blockage was introduced. Unlabeled probe melting analysis is developed on the basis of asymmetric PCR. After asymmetric PCR, a large number of superfluous single strands will hybridize with the unlabeled probe. So with the increasing in temperature, it will produce two types of melting curve. The part of curve in low melting temperature represents the region of probe and product.

**Table 1:** Sequences of primers and unlabeled probe for MCA and ARMS.

	Primer/probe		Sequence (5′-3′)
MCA	Forward		AGCTTTCTCACAAGCATTTGG
	Reverse		TGACACCTAGCTGTGATCCTG
	Probe		AAATTATGGAGTATGTTTCTGTGGAGACGAGA
ARMS	Outer primers	Forward	TCCTCAGAACGTTGATGGCAG
		Reverse	ATTGCTTTCCTTTTTCACAAGAT
	Specific primers	Wild type	GCATTTGGTTTTAAATTATGGAGTATATG
		Mutant	GTTTTACTTACTCTCGTCTCCACAAAA

Asymmetric PCR was performed in a 20 µl of reaction volume. The master mix contained: 10 µl Premix Taq® Hot Start Version (2×) (TaKaRa BIO, Shiga, Japan), 1 µl forward primer (0.5 µM), 1 µl reverse primer (5 µM), 1 µl unlabeled probe (5 µM), 1 µl SYTO® 9 dye (30 µM) (Invitrogen, Carlsbad, CA), 5 µl deionized distilled water (dd H_2_O) and 1 µl DNA template (15–25 ng/µl). The PCR mix was subjected to PCR in a Rotor-Gene® Q real-time platform (Qiagen, Valencia, CA). An initial denaturation was performed at 95°C for 2 min, followed by 50 cycles of 95°C for 30 sec, 58°C for 30 sec, and 72°C for 30 sec. After PCR, the products were heated to denaturation at 98°C for 2 min, followed by cooling down to 40°C for 2 min to facilitate the heteroduplex formation then melting slowly at 0.2°C/s from 50°C to 95°C. High resolution melting (HRM)-curve analysis was performed using Rotor-Gene® Q 1.7 software. For each assay we always include positive and negative controls (homozygous wild type, heterozygous, and homozygous mutant).

### Analytical sensitivity and reproducibility

The assay method was evaluated for sensitivity by running a serial dilution panel of homozygous HEL cell line DNA in wild type RPMI8226 cell line DNA at concentrations of 50%, 25%, 10%, 5%, 3% and 1% in order to evaluate the sensitivity of the methodology. HEL cell line DNA (homozygous mutant) and RPMI8226 cell line DNA(homozygous wild type) were analyzed repetitively by unlabeled probe MCA to confirm whether the melting curve was reproducible using both normalized and temperature-shifted difference plots.

### ARMS method

The allele-specific PCR testing for the JAK2 V617F mutation was performed as described previously [7]. This method used 2 primer pairs (Table 1) to specifically amplify the normal and mutant sequences with a positive control band in a single run. Amplifications were performed for 35 cycles with HotStar Taq® polymerase (Qiagen, Valencia, CA), an annealing temperature of 60°C, and standard amplification condition. Products were resolved on 3% agarose gels and visualized after staining with ethidium bromide.

### Sequencing

To verify the outcome by the MCA assay, we amplified ten samples as determined by unlabeled probe melting using the same primer pairs of MCA for sequencing analysis, in comparison with the JAK2 reference DNA sequence (GenBank accession number NT_008413.18). Amplicons were gel purified using the QIAquick® gel purification kit (Qiagen, Valencia, CA). DNA sequencing analysis was performed in PRISM® 310 genetic analyzer (Applied Biosystems, Foster City, CA). Given the limited sensitivity of sequencing, the purified products were cloned into the T-vector pMD-18T (TaKaRa BIO, Shiga, Japan) for the samples with discrepant results for sequencing of cloned insert.

### Validation of the method in three additional instruments at two cities

It has been reported that important discrepancies could be observed when one melting curve assay was used on different instruments [23], [24]. In order to know that whether the method can generate reliable and highly reproducible data on all commonly used real-time PCR instruments, we validated it with three additional routine real-time instruments. We thus compared the diagnostic accuracy of our method by analyzing the ten samples on three instruments, in two separate laboratories in Huashan Hospital of Shanghai and the First Affiliated Hospital of Dalian Medical University respectively. In both laboratories the detection was performed in a completely blind manner. After the amplification, melting analysis on the LightCycler® 480 (Roche, Mannheim, Germany) was performed in Dalian with high-resolution melting from 50°C to 85°C, 25 acquisitions/°C. The results were analyzed in the Gene Scanning mode of the LightCycler® 480 software package. ABI® 7500 (Applied Biosystems, Foster City, CA) and Mastercycler® ep realplex (Eppendorf, Hamburg, Germany) were used in Shanghai. Melting curve analysis was performed with the default settings on the ABI® 7500 System SDS 1.4 and Eppendorf Mastercycler® ep realplex 1.5 from 50°C to 85°C.

## Results

### Detection of the JAK2 V617F missense mutation detection in patients with PV using unlabeled probe MCA

After asymmetric PCR, the use of one primer in excess during asymmetric PCR leads to the overproduction of the single target strand and the probes anneal to these single-stranded products. Melting curves can be divided into two regions, representing the melting of probe/product and product/product strands, respectively. Different alleles result in different probe/product melting transitions based on the stability of the mismatches present. It is preferable to see these transitions by plotting the negative derivative (dF/dT) of fluorescence (F) versus temperature (T).

**Figure 1:**
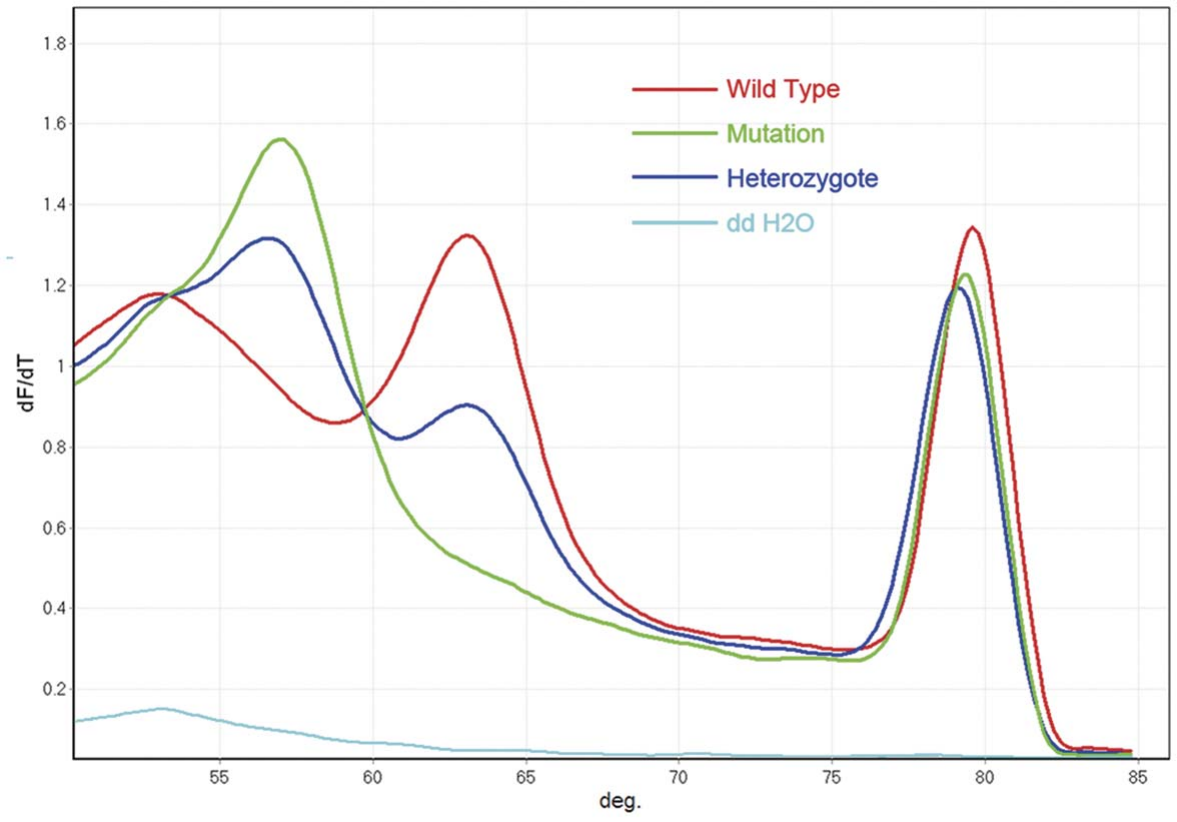
Unlabeled probe MCA on Rotor-Gene® Q. Deivative (dF/dT) plot of melting curve consists of two melting regions. The probe-target melting region lies in left side. Samples with the T allele (green) had a lower Tm of 57.3°C, while samples harboring the G allele (red) showed a Tm of 63.6°C. Therefore, the G/T heterozygous samples (blue) manifested both melting peaks of these alleles. Each genotype followed a unique path that distinguished itself from the others.

Probe-target melting for JAK2 V617F was observed between 50°C and 70°C. A perfectly matched probe-target hybrid has a characteristic melting temperature that is higher than a mismatched hybrid. A closer examination of the region of probe melting showed that samples with the T allele had a derivative melting peak at 57.3°C, whereas samples harboring the G allele showed a melting peak at 63.6°C (Figure 1). The heterozygous samples showed two peaks, one at each temperature representing the combination of both alleles. Therefore, a single probe was able to recognize all three genotypes within the given sample set. Cytogenetically, patients with polycythemia vera may be heterozygous or homozygous for the JAK2 V617F mutation. It would be difficult to distinguish a “true” heterozygous state at the single-cell level for a clonal population of somatically mutated cells in a background of wild type cells from peripheral blood, so none of the current assays performed would be appropriate for reliably establishing true heterozygosity or homozygosity for the mutation. Thirty-seven out of 40 patients (92.5%) were positive for the presence of JAK2 V617F mutation.

### Analytical Sensitivity

**Figure 2:**
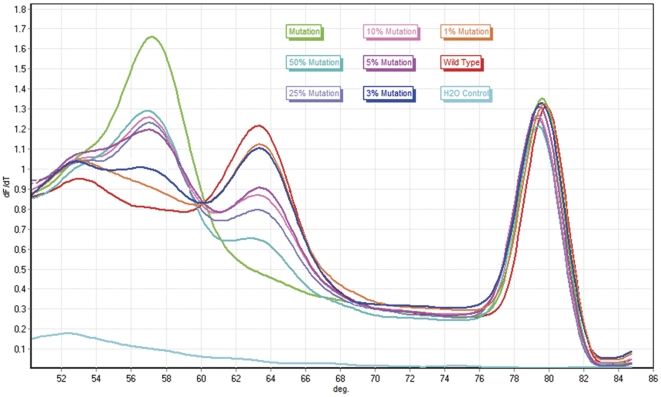
Detecting sensitivity of unlabeled probe MCA on Rotor-Gene® Q. For standard heterozygous samples containing over 3% (blue) T allele (mutation), there was a shape melting peak at Tm of the probe-mutation intermediates, which could be easily distinguishable from that of the wild type melting transition.

In order to evaluate the sensitivity of our method, we mixed wild type DNA with different concentrations (1%, 3%, 5%, 10%, 25%, and 50%) of mutant DNA. In this study, up to 3% of the JAK2 V617F mutation was successfully detected in patients with PV using unlabeled probe MCA (Figure 2).

To test intra- and inter-assay CV of the assay, we compared the Tm of derivative melting peaks for T and G alleles obtained from 20 results of the same assay and 4 independent assays carried out on different days. At the level of 5%, intra- and inter-assay CVs ranged from 3.14%/3.55% and 1.72%/1.29%, respectively.

### Detection of the JAK2 V617F missense mutation detection in patients with PV using the ARMS assay and sequencing

**Figure 3:**
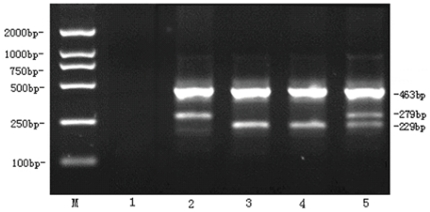
ARMS assay for JAK2 V617F. Of tracks for each sample, bands in 229-bp suggested the existence of the wild type allele, while a mutant allele was indicated by the presence of a band in 279-bp. The 463-bp product served as a control of amplification. M, 2000 bp DNA ladder; 1, dd H_2_O water control; 2, HEL cell line DNA as JAK2 V617F homozygous control; 3, RPMI82264 cell line DNA as wild type homozygous control; 4, Patient sample with no JAK2 V617F mutation; 5, Patient sample harboring wild type/JAK2 V617F positive heterozygote.

In the ARMS assay, outer primers flank the mutation region and create a 463-bp control product. A wild type-specific forward primer pairs with the reverse control primer to form a 229-bp product; if JAK2 V617F is present, the mutation-specific reverse primer will form a 279-bp product with the forward control primer (Figure 3). Samples tested by the ARMS method revealed the exact same results, indicating that our MCA achieves 100% accuracy. Twenty randomly selected samples including eighteen JAK2 V617F positive samples and two JAK2 V617F negative samples identified by MCA were subject to direct sequencing. There were two discrepant results, negative in the sequencing but positive in the unlabeled probe MCA. After T-A cloning, sequences of cloned PCR products revealed the mutation in the two samples, suggesting the better detection sensitivity and diagnostic efficiency of the unlabeled probe MCA.

### Validation of the method in three additional instruments

**Figure 4:**
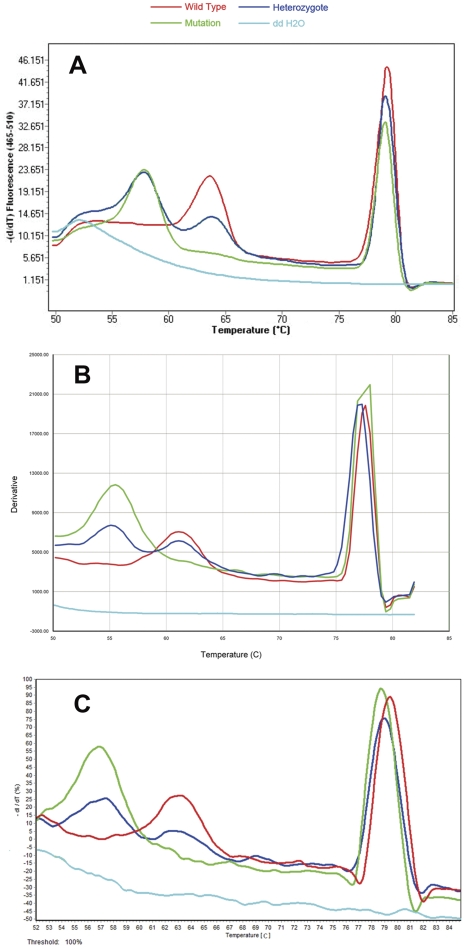
Validation of the unlabeled probe MCA on three different real-time instruments. LightCycler® 480 (Figure 4A), ABI® 7500 real-time PCR system (Figure 4B) and Mastercycler® ep realplex (Figure 4C). The wild type homozygote (red) presented a probe melting peak of 63.8°C (LightCycler®), 61.7°C (ABI® 7500) and 63.4°C (Mastercycler®), while the JAK2 V617F homozygote (green) produced a probe melting peak of 57.9°C (LightCycler®), 55.6°C (ABI® 7500) and 57.5°C (Mastercycler®). A wild type/mutation heterozygote showed both melting peaks of wild type and mutation.

The validation of the method was completed by analyzing ten samples bearing different mutated sequences (8 JAK2 V617F positive samples and 2 JAK2 V617F negative samples) in other two different laboratories in Shanghai and Dalian, which had not participated in the establishment of the unlabeled probe assay methodology and were equipped with different real-time PCR systems. Although shifting temperatures and shapes of curves were somewhat different among the three instruments, results were undoubtedly similar in their interpretation. Both laboratories perfectly identified every mutated sample, without any change in the experimental protocol confirming this unlabeled probe MCA as a robust and efficient method even in an inter-laboratory fashion. The sample with the G allele generated a melting peak of 63.8°C, 61.7°C and 63.4°C for LightCycler® 480, ABI® 7500 real-time PCR system and Mastercycler® ep realplex respectively, whereas sample with the T allele presented a melting peak of 57.9°C, 55.6°C and 57.5°C for LightCycler®, ABI® 7500 and Mastercycler® respectively (Figure 4).

## Discussion

The JAK2 V617F mutation, first described in 2005, has become an important diagnostic criterion in Philadelphia chromosome negative myeloproliferative diseases, especially in polycythemia vera (PV) [1]–[4]. In accordance with this, it has been recently included in the World Health Organization (WHO) diagnostic criteria for Philadelphia chromosome negative myeloid neoplasms [5].Furthermore, pharmacological JAK2 inhibitors are a new type of treatment for myelofibrosis and potentially other types of myeloid neoplasms as well [25]–[27], reinforcing the need for a convenient tool to detect this mutation.

In general, the PCR-RFLP [2], ARMS [7] and sequencing [10], [11] are common alterations for the detection of JAK2 mutation because they are suitable for small-scale diagnostic procedures in individual hospital laboratories. However, both ARMS and RFLP retain some technical challenges that must be resolved, especially regarding the long turnaround time, low throughput, poor sensitivity, and both methods are prone to DNA contamination. Methods such as sequencing are also able to identify SNPs, but require the purchase and setting up of special sequencing facilities and additional sample preparation reagents and steps. Furthermore, sequencing was unable to detect mutations below an approximately 20% clonal load [28]. For this reason, several kinds of different methodologies to analyze the amplicons have been developed. Lay et al. [9], Ochsenreither et al. [29] and Olsen et al. [30] described rapid PCR with fluorescently labeled oligonucleotide hybridization probes as a molecular diagnostic method to detect the JAK2 V617F missense mutation. However, these studies used expensive reagents such as the flurorophores, resulting in an increase in cost compared with any other analysis.

High-resolution melting was recently introduced as a new technique to genotype SNPs within small amplicons [31]–[33], which was enabled by novel saturation dyes and high-resolution instruments. Rapado et al. [34] developed the HRM analysis for screening JAK2 V617F mutation in patients with myeloid neoplasms. Although HRM analysis is sensitive, accurate and elegant, it requires expensive instrumentation. Because of their cost and complexity, these techniques might not be used routinely by all laboratories.

Recently, an unlabeled probe assay with saturation dye for genotyping was described [14]–[16]. This approach uses asymmetric PCR in the presence of the DNA intercalating dye LC Green® with an unlabeled probe specific to the SNP of interest. Although this method is superior to conventional HRM analysis in the identification of many small insertions or deletions and some class 3 and 4 SNPs (∼4% of human SNPs), differentiating between the two possible homozygotes can be problematic when probes are not used [35].

In this study, we developed a melting assay with unlabeled probe for detection of the JAK2 V617F point mutation for use in a routine diagnostic setting on Rotor-Gene® Q. Unlabeled probes are usually approximately 30–40 bps in length and are blocked at their 3′-end to prevent extension [22], [23], so a high sensitivity rate can be obtained. By introducing an unlabeled probe covering the SNP, the different genotypes can be clearly distinguished. This method achieved 100% accuracy compared with ARMS results. Each assay method was evaluated for sensitivity by running a serial dilution panel of homozygous V617F HEL cell line DNA in wild type DNA at concentrations of 1%, 3%, 5%, 10%, 25% and 50%. Our laboratory defined a melting peak at 3% as a weak positive result, although the threshold for a weak positive with this assay may vary between laboratories.

Previous authors utilizing Real-time PCR analysis for JAK2 V617F detection have published assays showing variable sensitivity. Lay et al. [9] and Olsen et al. [30] used real-time PCR methods and produced tests with analytical sensitivities of 5%. According to the study of Qian et al. [36], up to 5% of the JAK2 V617F mutation was also successfully detected in patients with myeloid neoplasms using HRM analysis. The analytic sensitivity of our assay (3%) is at least equivalent to thet currently documented qualitative real-time PCR test for JAK2 V617F. The method had melting curves with robustly reproducible discernible differences (within-run and between- run) down to the 5% level.

According to the literature [20]–[22], closed-tube genotyping with unlabeled oligonucleotide probes can be performed on several types of instruments. We validated the method as reproducible and transferable on three different instruments with varied thermal melt resolution. We have shown that unlabeled probes can be used to accurately determine genotype from standard melting curves even on low resolution machines (ABI® 7500 and Mastercycler® ep realplex). In addtion, overcoming the drawbacks of SYBR Green I, we employed the saturate interacting dye SYTO® 9 [37] to support PCR amplification in a high concentration of dye and produce robust DNA melting curves that are not affected by DNA concentration [38], [39], thus improving the precision of melting temperature of the dissociation peak and its signal strength.

In summary, we developed a novel unlabeled probe MCA that utilizes asymmetric PCR followed by melting curve analysis to detect the JAK2 V617F mutation. Either conventional real-time PCR instruments or more economic, low resolution instruments can be used. High-resolution analysis is not necessary, indicating the wide applicability of our method in clinical laboratories.

Additional benefits of the closed-tube versus the open-tube assay include the elimination of labor costs due to manual post-PCR analysis and prevention of cross contamination. Nevertheless, the unlabeled probe method presented in this study can be applied to almost every real-time PCR platform with melting curve function, and is a high throughput method which is much more suitable for routine laboratory diagnosis. This method obtains reproducible results in both inter-laboratory and inter-instrument situations.
